# Effects of *Lactiplantibacillus plantarum* on Metabolites and Flavors in Synthetic Microbiota During Baijiu Fermentation

**DOI:** 10.3390/foods14010031

**Published:** 2024-12-26

**Authors:** Chunyue Yan, Xurui Chen, Quan Liu, Tengyu Xu, Qian Zhang, Xueli Jin, Bei Liao, Xiong Chen, Xin Li

**Affiliations:** 1 Cooperative Innovation Center of Industrial Fermentation (Ministry of Education and Hubei Province), Key Laboratory of Fermentation Engineering (Ministry of Education), Hubei Provincial Key Laboratory of Industrial Microbiology, School of Life and Health Sciences, Hubei University of Technology, Wuhan 430068, China; yanchunyue26@163.com (C.Y.); chen0203092020@163.com (X.C.); 15871452919@163.com (Q.L.); 15827453028@163.com (T.X.); 18095010330@163.com (Q.Z.); 15895839198@163.com (X.J.); chenxiong@mail.hbut.edu.cn (X.C.); 2 Angel Yeast Co., Ltd., Yichang 443000, China; liaobei@angelyeast.com

**Keywords:** microorganism of food fermentation, *Lactiplantibacillus plantarum*, sensory evaluation, microbial interactions, fermentation performance, synthetic chassis microbiota

## Abstract

The distinctive flavor and aroma of Chinese baijiu are closely linked to the microorganisms involved in the fermentation process. *Lactiplantibacillus plantarum*, a dominant species in the fermentation of Chinese baijiu, has become a prominent research focus. In this study, we selected well-characterized pure cultures of microorganisms to construct diverse chassis microflora. The primary objective was to investigate the effects of *L. plantarum* on the fermentation process of Chinese baijiu and its association with metabolites produced by different chassis microflora. Our results demonstrated that the concentrations of ethyl lactate and other volatile aromatic compounds increased in all fermentation protocols where *L. plantarum* was added. The addition of *L. plantarum* also significantly increased the concentration of total organic acids, particularly lactic acid, which rose by 17 to 123 times. Furthermore, *L. plantarum* helped maintain the stability of ethanol concentration during the middle and late stages of fermentation. Notably, among the three different chassis microbial fermentation protocols involving *L. plantarum*, the protocol with the highest microbial diversity exhibited a greater capacity to produce lactic acid (1.56 ± 0.19 mg/g), ethanol (5.74 ± 0.47 mg/g), and reducing sugars (6.39 ± 0.31 mg/g). These findings provide valuable insights into the potential of *L. plantarum* for modulating the flavor of Chinese baijiu.

## 1. Introduction

Chinese baijiu, one of the most popular alcoholic beverages, with a rich history of brewing and consumption dating back thousands of years, is recognized alongside brandy, whisky, vodka, gin, and rum as one of the world’s most renowned distilled spirits [1]. Chinese traditional baijiu is made primarily from sorghum, wheat, rice and other grains, and Daqu, Xiaoqu, Bran Koji and active dry yeast are used as the main fermentation agents. These ingredients are processed through cooking, saccharification, fermentation, distillation, and storage to produce pure Chinese baijiu [2].

The aroma and taste of Chinese baijiu is one of the main reasons influencing people’s consumption [3]. Aroma-active compounds that are soluble in Chinese baijiu significantly determine and influence its taste and quality, and these compounds give alcoholic beverages a distinctive fruity, floral or off-flavor [4,5]. In previous studies, gas chromatography-mass spectrometry (GC-MS) coupled with gas chromatography-olfactometry (GC-O) has proved to be an effective method for characterizing aroma-active compounds in baijiu [6,7]. The most classical and commonly used sensory technique in baijiu is quantitative descriptive analysis (QDA), which not only describes the sensory characteristics of the product, but also distinguishes sensory differences between different samples [8,9]. However, the process of sensory flavor analysis of baijiu mainly depends on the personal experience of the sommelier, which is subjective and inconsistent. In order to objectively evaluate the quality of high-quality baijiu and improve yield, it is necessary to characterize the flavor profiles of different baijiu and identify their corresponding key compounds [10]. Therefore, there is a need for instruments with higher separation capabilities to obtain an accurate profile of volatile organic compounds (VOCs) in alcoholic beverages and to effectively identify each VOC. Two-dimensional gas chromatography-mass spectrometry (GC×GC-MS) is a well-established technique for identifying and performing quantitative analysis of VOCs in fermented beverages [11].

Quantitative analyses have demonstrated that *Lactobacillus* spp. were the most abundant bacterial group in the fermentation process of Chinese baijiu jiupei [12]. Regardless of the flavor profile of Chinese baijiu, *Lactobacillus* spp. remained the most dominant bacteria in the later stages of fermentation [13,14]. The organic acids produced by lactic acid bacteria fermentation lower the pH of the entire fermentation system, affecting the growth and metabolism of other microorganisms in the microflora during the fermentation of Chinese baijiu [15,16]. Nevertheless, *Lactobacillus* species possess antacid genes such as *argR*, *aspA*, and *ilvE*, which enable their survival during the later stages of fermentation, characterized by high acid and ethanol concentrations [17]. *Lactobacillus* bacteria, as the dominant microorganisms in the brewing process of Chinese baijiu, not only produce lactic acid through homolactic fermentation but also generate acetic acid via heterolactic fermentation [18]. Acetic and lactic acids can subsequently serve as substrates for the synthesis of butyric and caproic acids [19]. These acids and alcohols undergo esterification to form important esters [20,21,22]. Among the various flavor compounds, esters were found to be the most significant substances influencing the flavor of Chinese baijiu. Different concentrations and ratios of the four ethyl esters—ethyl acetate, ethyl lactate, ethyl butyrate, and ethyl caproate—could produce distinct flavor profiles in Chinese baijiu [21]. From the above, it was evident that lactic acid bacteria played a crucial role in the flavor formation of Chinese baijiu. However, the other biochemical effects of artificial mixed fermentation using pure species of *Lactobacillus* and pure strains of chassis microorganisms on Chinese baijiu remain unknown.

Traditionally, Chinese baijiu was produced in a relatively open environment through solid-state fermentation, where a diverse array of complex microorganisms interacts with the grain. Different chassis microflora can significantly influence the aroma of Chinese baijiu by modulating metabolic pathways [23]. For example, *Aspergillus* species, which exhibit high glucose amylase and α-amylase activity, play a crucial role as key functional microorganisms by providing essential nutrients for the microbial community during the baijiu brewing process [24]. *Saccharomyces cerevisiae* and *Saccharomycopsis fibuligera* are the primary yeasts responsible for alcohol production and ester formation, respectively, during the fermentation process of Chinese baijiu. Additionally, *Thermophilic actinomycetes*, *Bacillus licheniformis*, and *Lactiplantibacillus plantarum* are all important bacterial species involved in the fermentation process of Chinese baijiu [25,26,27,28,29,30]. The richness of raw materials, the co-fermentation of multiple strains, and the complexity of the process mean that the mystery of Chinese baijiu’s flavor and texture has not yet been fully explored. Compared with natural inoculation brewing, artificial pure-culture mixed brewing could provide a clearer background of the microbial community [31].

*L. plantarum*, which belongs to the *Lactobacillus* spp., has been proven to be safe for use in food brewing and exhibits good brewing characteristics [28]. Currently, most research on Chinese baijiu focuses on the evolution of open microbial communities, microbial sources, and microbiota interactions [32,33]. However, there are few reports on the effect of the interaction of *L. plantarum* with different synthetic chassis microflora on the fermentation of Chinese baijiu. In this study, we chose *A. oryzae*, *S. fibuligera*, and *S. cerevisiae* as the base microorganisms for the fermentation of Chinese baijiu. On the basis of these chassis microorganisms, *T. actinomycetes* and *B. licheniformis* were added sequentially to form different synthetic microbiota fermentation combinations.

By conducting a comparative analysis of fermentation samples for various metabolites (esters and other volatile flavor compounds, lactic acid, ethanol, organic acids), α-amylase and glucoamylase activity, and other physico-chemical indices (reducing sugar, pH), we revealed the influence of *L. plantarum* on the fermentation outcomes in different chassis microbial environments of Chinese baijiu. We also examined the differences in the metabolism of various microbiota assemblages in the presence of *L. plantarum*. These results not only provided a solid theoretical basis for the application of *L. plantarum* in the brewing of Chinese baijiu but also offered valuable guidance for the development of new aroma types of Chinese baijiu.

## 2. Materials and Methods

### 2.1. Selection and Cultivation of Strains

In this study, we employed six microorganisms: two strains of yeast (*S. fibuligera* YC-26, *S. cerevisiae* BS-19), one strain of mold (*A. oryzae* M-08), and three strains of bacteria (*B. licheniformis* NB-06, *L. plantarum* YP-83, and *T. actinomycetes* MC-34). The microorganisms, isolated from Baijiu fermentation pellets, are kept in the laboratory of the Functional Yeast and Fermentation Microbiology Research Group. The strains were maintained in glycerol at a final concentration of 50% (*v*/*v*) at −80 °C. For the activation of *S. cerevisiae* BS-19, glycerol stocks were transferred to 30 mL of YEPD solid medium and incubated at 30 °C in a temperature-regulated incubator (HNHWS-II-250, Tianjin Honour Instrument Co., Ltd., China) until the logarithmic growth stage was reached [34]. To activate *S. fibuligera* YC-26, glycerol stocks were inoculated into 100 mL of 1/3 YEPD liquid medium and cultured in a temperature-regulated shaker at 30 °C (200 r/min) until reaching the logarithmic growth phase [34]. *L. plantarum* YP-83 was activated by inoculating glycerol stocks into 30 mL of MRS solid medium and incubated at 37 °C in a temperature-controlled incubator until the logarithmic growth phase was attained. *T. actinomycetes* MC-34 was activated by transferring glycerol stocks to 100 mL of modified Gauchy’s liquid medium and culturing at 45 °C (150 r/min) in a constant temperature shaker until the logarithmic growth stage. For activation, glycerol stocks of *B. licheniformis* NB-06 were transferred to 70 mL of LB slant solid medium and incubated at 37 °C in a temperature-regulated incubator until the logarithmic growth phase was achieved. Glycerol stocks of *A. oryzae* M-08 were transferred to 30 mL of potato dextrose agar (PDA) medium and grown at 30 °C under constant temperature conditions until spore formation [34].

The yeast and bacterial strains were enumerated using the dilution plating method, and the spores of *A. oryzae* M-08 were counted using the hemocytometer method [35].

### 2.2. Fermentation Protocols and Inoculum Amounts

Following inoculation with microorganisms, the sorghum was incubated at 30 °C in a constant temperature incubator. The sorghum was rehydrated to its original weight every 4 d and thoroughly mixed. The fermentation lasted 15 days, and each experimental group was replicated three times. The experimental protocol and inoculum amounts are detailed in Table 1. In this study, six fermentation protocols (CK1, CK2, CK3, F1, F2, F3) were established. The control groups consisted of three fermentation protocols with different microbial diversities (CK1, CK2, CK3). *L. plantarum* YP-83 was added to all three control groups to establish three experimental groups (F1, F2, F3). The total bacterial inoculum (*B. licheniformis* NB-06, *L. plantarum* YP-83, and *T. actinomycetes* MC-34) and total fungal inoculum (*S. fibuligera* YC-26, *S. cerevisiae* BS-19) was maintained at a 1:1 ratio (excluding *A. oryzae* M-08). In all six fermentation protocols, *A. oryzae* M-08 was added at a mass ratio of 0.5 (OD_600_ per gram of raw material). The final inoculum was based on the number of viable bacteria added to the sorghum medium, as shown in Table 1.

### 2.3. Sample Collection and Raw Material Handling

Thirty grams of sorghum were weighed and subjected to sterilization in a 250 mL Erlenmeyer flask at 121 °C for 20 min using a moist heat sterilizer (YXQ-LB-50SII, Shanghai Boxun Industry and Commerce Co., Ltd. Medical Equipment Pactory, Shanghai, China). Once sterilized, the samples were cooled to ambient temperature and 10 mL of deionized water was poured in, followed by thorough mixing for a second sterilization. Inoculation was performed on the second day after the completion of sterilization. The weight of each bottle of sorghum medium was approximately 70 ± 0.3 g, with the moisture content controlled at 57.3% ± 0.1%.

At days 0, 2, 4, 6, 9, 12, and 15 of fermentation, samples were taken. The combined weight of an Erlenmeyer flask and the sorghum was measured, then 100 mL of sterile water was added. After this, the Erlenmeyer flasks were placed in a constant temperature shaker (HNYC-202T, Tianjin Honour Instrument Co., Ltd., Tianjin, China) at 30 °C and 200 r/min for 45 min.

After this, 45 mL of the samples to be tested was removed and transferred to a 50 mL centrifuge tube. The supernatant was gathered for further use after centrifuging the samples at 4529× *g* for 5 min at ambient temperature. On the same day, pH was measured and glucoamylase, α-amylase, and reducing sugar samples were tested. For a maximum of 5 d, flavor matter, ethanol, and organic acid samples were stored in a refrigerator at −20 °C.

### 2.4. Determination of the Viability of Two Enzymes and Other Physico-Chemical Parameters

For the detection of α-amylase and glucoamylase activity and other physico-chemical indicators such as ethanol, lactic acid, reducing sugar, pH, and organic acids, we used the methods described in previous assays [35,36]. The concentration of ethanol was determined using the biosensor method, the concentration of reducing sugar was determined using the DNS method, the pH value was measured using a pH meter (FiveEasy Plus FP20, Mettler-Toledo International Inc., Greifensee, Switzerland), and gas chromatography (GC) was employed to analyze the organic acids (acetic acid, propionic acid, butyric acid, isobutyric acid). A detailed description of these detection methods can be found in previous reports [35].

The amount of enzyme that releases 1 mg of reducing sugar per hour under the assay conditions was defined as one unit of saccharifying enzyme activity. The amount of enzyme that causes the disappearance of 1 μg of iodine-bound starch material per minute at 50 °C and pH 7.0 was defined as one unit of α-amylase activity.

### 2.5. Analysis of Volatile Compounds by GC-MS

Using a GC-MS TQ8050 NX system (Shimadzu, Kyoto, Japan) equipped with an InertCap WAX chromatographic column (30 m × 0.25 mm × 0.25 µm, GL Sciences Inc., Tokyo, Japan), headspace solid-phase microextraction coupled with gas chromatography–mass spectrometry (HS-SPME-GC–MS) was performed [37].

Initially, 5 mL of fermentation samples were placed in 20 mL headspace vials and mixed with 1.5 g of NaCl. Each vial received a 4-methyl-1-pentanol internal standard solution (1.98 μg/L). The headspace vials containing the samples were placed in a thermostatically heated magnetic stirrer set at 45 °C and stirred at 500 rpm for 30 min (DF-101D, Gongyi Yuhua Instrument Co., Ltd., Gongyi, China). Subsequently, SPME fiber (50/30 µm, DVB/CAR/PDMS, Supelco, Inc., Bellefonte, PA, USA) was inserted into the headspace vial, and the extraction proceeded with continued stirring for an additional 30 min. Upon completion of the extraction, the SPME fibers were desorbed in a gas chromatography injector at 250 °C for 5 min.

Note: SPME fibers need to be high temperature aged for 5 min before use to remove impurities; the fibers should be properly cleaned and stored after each use to maintain their reliability for following uses.

In electron ionization (EI) mode at 70 eV, mass spectra were acquired over the range of *m*/*z* 50–450. The retention index (RI) was determined using a mixture of n-alkanes (C8-C40), and compounds were tentatively identified by their retention time, mass spectra, and a similarity threshold of 85%, using the NIST 14 library as a reference (Note: Under all experimental conditions, the RI difference does not exceed 0.005). Each experiment was repeated three times, and where possible, compound identification was confirmed by comparing with authentic standards. To rapidly screen for and determine the concentrations of target flavor compounds in the samples, the single-point calibration method was employed.

The formula for calculating the concentration of flavor compounds is as follows:

(1) $$C=(\frac{\;A}{\;B})\times D$$

where *A*: sample peak area; *B*: standard peak area; *C*: Sample concentration; *D*: Standard solution concentration (1.98 μg/mL).

### 2.6. Sensory Evaluation Standards

Twelve judges, including 6 men and 6 women from Hubei University of Technology, conducted the sensory evaluation. The evaluators were trained in a sensory room maintained at 25 °C ± 2 °C and had more than 2 yr of sensory evaluation experience. The terminology for describing the flavor profile was based on the standard GB/T 10221-2021 [38]. “Sensory Analysis Terminology” and was jointly developed by the evaluators after assessing various samples and reference materials [39,40].

Samples were evaluated after thorough stirring, having been prepared at a supply temperature of 30 °C ± 2 °C. The common descriptive terms used in the evaluation were adopted as the result of the flavor assessment. Sensory attributes such as fruit aroma, floral aroma, cereal aroma, herbaceous aroma, acidity, sweetness, nail polish aroma, rancid flavor, astringency, full body, and persistence were assessed. A six-point scale was used for scoring the fermentation sample profiles:

0: Imperceptible

1–2: Slight

3–4: Mid-range

5–6: Intense

7–8: Very intense

The computational formula for the scores was as follows:

(2) $$Score=\frac{\sum Individual\;Scores}{Number\;of\;Evaluators}$$

### 2.7. Statistical Analysis

Using the external standard method and based on the standard curve, the concentrations of sugar and organic acid were quantitatively analyzed. Each experiment was repeated three times, and statistical analysis was conducted using IBM SPSS Statistics 23 (International Business Machines, Armonk, NY, USA)

Differences were considered significant at *p* < 0.05 and highly significant at *p* < 0.01. Origin 2019b (OriginLab Corporation, Northampton, MA, USA) was used for plotting, PCA, and generating cluster heatmaps.

## 3. Results and Discussion

### 3.1. L. plantarum Contributes to the Accumulation of Esters

Chinese baijiu detected more than 1870 volatile flavor compounds and 48 non-volatile organic acids and polyhydroxy compounds [1]. Among them, organic acids account for 14–16%, alcohols for 12%, carbonyl compounds for 6–8%, and other compounds for 4–8% [41]. Ester compounds account for more than 60% of the total mass of all flavor substances and play a crucial role in influencing the flavor of Chinese baijiu, significantly contributing to its aroma [42].

**Figure 1:**
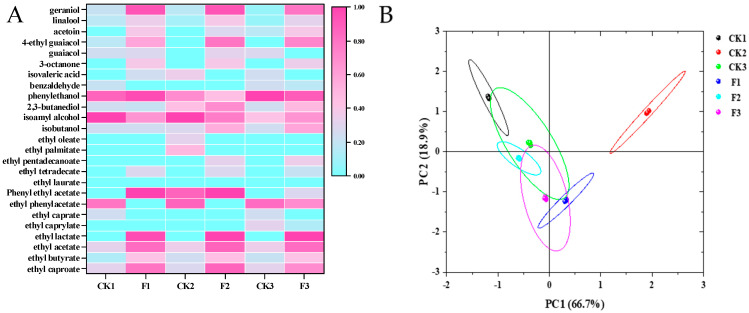
(**A**): Heatmap of volatile aroma compound concentrations in experimental (F1, F2, F3) and control (CK1, CK2, CK3) groups. (**B**): PCA graph of experimental (F1, F2, F3) and control (CK1, CK2, CK3) groups. Same color labels indicate parallel experiments.

In this study, a total of 25 volatile flavor components were detected across the six fermentation protocols (including 13 esters, 4 alcohols, 1 aldehyde, 2 acids, 1 ketone, 2 terpenes and 2 phenolic compounds) (Figure 1A). The heatmap depicts the general volatile profile through a color gradient in terms of the raw R value. Different fermentation protocols normalized the color scale from 0 (light blue) to 1 (rose red), representing volatile abundance from low to high (Figure 1A), and the color of low-concentration substances was not obvious in the clustered heatmap.

The F1, F2, and F3 groups showed a significant change in the metabolic distribution of flavor substances compared to the CK1, CK2, and CK3 groups, consistent with the findings of a previous study [43]. The concentrations of four important esters-ethyl lactate, ethyl acetate, ethyl butyrate, and ethyl hexanoate-were significantly increased (Figure 1A). Ethyl lactate was not detected in any of the three control groups (CK1, CK2, CK3) without *L. plantarum* in this study. However, it was detected in the three experimental groups (F1, F2, F3) containing lactic acid bacteria, yeasts, and molds at concentrations of 200.00 ± 3.42 μg/g (F1), 210.00 ± 3.27 μg/g (F2), and 230.00 ± 2.11 μg/g (F3). Previous reports have indicated that the synthesis of ethyl lactate in baijiu primarily occurs through the esterification reaction between lactic acid and ethanol, which requires the joint participation of lactic acid bacteria, yeasts, and molds [21]. Various lactic acid bacteria (e.g., *Lactobacillus*, *Weissella*, *Pediococcus*, and *Leuconostoc*) and yeast communities provide lactic acid and ethanol as substrates, while some yeasts and molds secrete esterases to catalyze the reaction. The synthesis of ethyl lactate in this study follows a similar pattern to previous findings [21].

Additionally, the concentrations of isovaleric acid, phenylethanol, isoamyl alcohol, and 2,3-butanediol showed significant decreases in this study, while the concentrations of other esters, such as phenylethyl acetate, increased. This phenomenon may be related to the previous report that acids and alcohols can serve as precursors to esterify and produce esters under enzymatic catalysis [21]. *L. plantarum* not only plays a crucial role in the synthesis of ethyl lactate but also contributes positively to the synthesis of other esters. To further visualize the differences between the three control groups (CK1, CK2, CK3) and the three experimental groups (F1, F2, F3), a PCA plot was generated using the concentrations of the 23 volatile compounds as the dataset (Figure 1B). The first two components in Figure 1B account for 85.6% of the total variance (66.7% for PC1 and 18.9% for PC2). The closer the data points are to one another in the figure, the larger the area of intersection of the confidence ellipses, which indicates a greater correlation between the two fermentation protocols. The confidence ellipses for the six fermentation protocols were more dispersed, with F1 and CK1, F2 and CK2, and F3 and CK3 located in different quadrants with minimal intersection. This suggests a very small correlation between the control and experimental groups, indicating that the addition of *L. plantarum* during fermentation had a significant impact on the accumulation of volatile flavor compounds.

In addition, we found that F1 and F2 in the experimental group were located in different quadrants in Figure 1B, and their confidence ellipses did not intersect. However, the confidence ellipses of F3 intersected with those of F1 and F2 to a certain extent. This suggests that, in the different experimental groups where *L. plantarum* was added, the interactions between different microorganisms could produce interesting differences in the fermentation results.

### 3.2. Sensory Evaluation of Baijiu Fermentation Samples by L. plantarum

**Figure 2:**
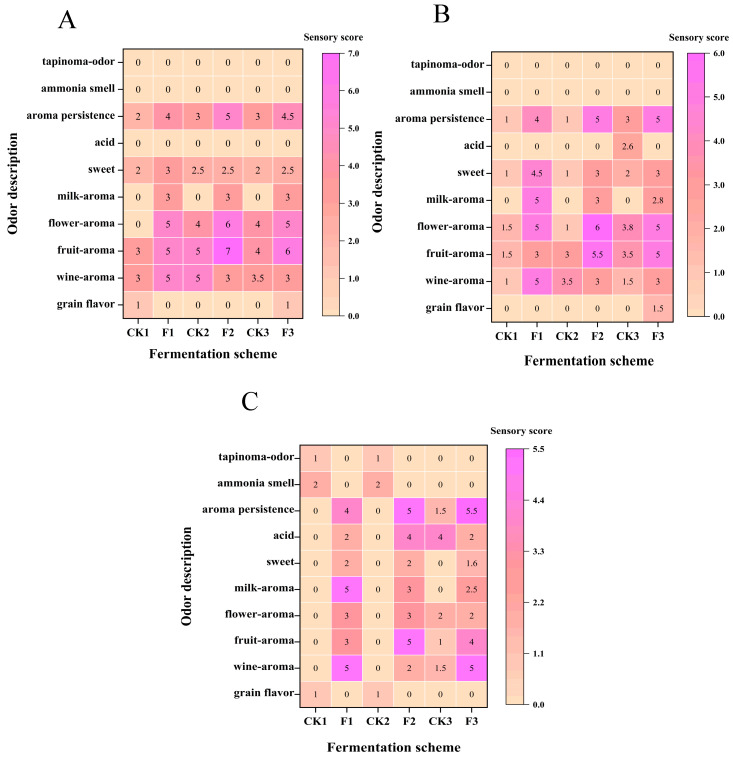
(**A**–**C**) show the heat maps of sensory evaluation scores for the experimental and control groups at the early (Day 2), middle (Day 8), and late (Day 15) stages of fermentation. Lighter colors represent lower scores, and darker colors represent higher scores. Numbers in the heat maps indicate the scores.

In Figure 2A–C, it is observed that the fruity, floral, creamy, and aroma persistence scores of the experimental groups (F1, F2, F3) were significantly higher than those of the control groups (CK1, CK2, CK3). In the middle stage of fermentation (Figure 2B), the floral aroma score for CK2 was only 1 point, while the floral aroma score for F2 with *L. plantarum* was 6 points. Throughout the early, middle, and late stages of fermentation, the control groups (CK1, CK2, CK3) showed no milk aroma, whereas the experimental groups (F1, F2, F3) exhibited moderate to strong milk aromas (early stage: F1 3 points, F2 3 points, F3 3 points; middle stage: F1 5 points, F2 3 points, F3 2.8 points; late stage: F1 5 points, F2 3 points, F3 2.5 points) (Figure 2B,C). It is speculated that certain esters contribute to the milk flavor in the experimental groups.

From Figure 2C, it is evident that CK1 and CK2 lacked wine and floral aromas in the late stage of fermentation and exhibited negative flavors such as ammonia odor (CK1: 2 points, CK2: 2 points) and rancidity (CK1: 1 point, CK2: 1 point). After the addition of *L. plantarum*, the ammonia odor and rancidity in F1 and F2 disappeared, and pleasant flavors such as fruity, wine, and floral aromas emerged. The addition of *L. plantarum* enriched the pleasant flavors (e.g., fruity, floral) in the fermentation system and reduced the generation of negative odors through metabolic regulation.

It is also worth noting that the floral aroma (Day 2: F1 5, F2 6, F3 5 points; Day 15: F1 3, F2 3, F3 2 points) and fruit aroma (Day 2: F1 5, F2 7, F3 6 points; Day 15: F1 3, F2 5, F3 4 points) of the three experimental groups tended to diminish as fermentation progressed. Additionally, the milk aroma of F3 (Day 2: 3 points; Day 8: 2.8 points; Day 15: 2.5 points) also decreased over time (Figure 2A–C). The interactions between microorganisms influence the survival of microbial communities, and this phenomenon suggests that controlling fermentation time could lead to better fermentation outcomes.

### 3.3. The Addition of L. plantarum Had Positive Effects on Increasing Lactic Acid Concentration and Maintaining the Stability of Ethanol Concentration

**Figure 3:**
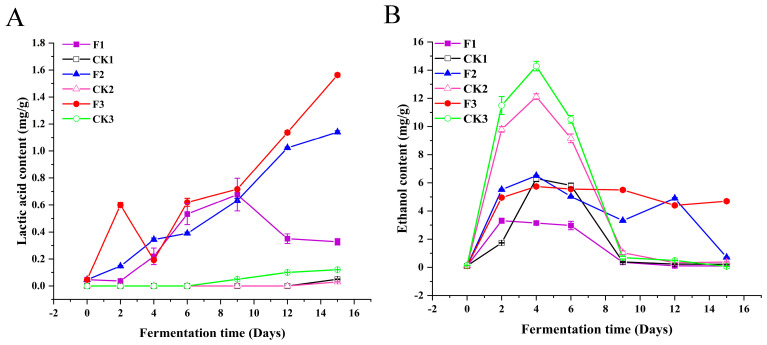
(**A**): Lactic acid concentration trends in experimental (F1, F2, F3) and control (CK1, CK2, CK3) groups over 15 days. (**B**): Ethanol concentration trends in experimental (F1, F2, F3) and control (CK1, CK2, CK3) groups over 15 days.

Ethyl lactate, an important flavor compound in most Chinese baijiu, is produced by the esterification reaction between lactic acid and ethanol [44]. Ethyl lactate enhances the sweetness and mellowness of Chinese baijiu [23]. Changes in the concentrations of lactic acid and ethanol during fermentation can affect the concentration of ethyl lactate. Ethanol is also a key contributor to the primary flavor of Chinese baijiu [1]. Therefore, both lactic acid and ethanol are important metabolites that influence the quality of Chinese baijiu. The changes in lactic acid and ethanol concentrations in the control and experimental groups are shown in Figure 3A,B.

Figure 3A,B show the trends of lactic acid and ethanol concentrations over the 15-day fermentation period. As shown in Figure 3A, the first 9 days of fermentation saw almost no lactic acid production in the control groups (CK1, CK2, CK3), with trace amounts beginning to appear only in the later stages. However, the lactic acid concentration in the control groups remained negligible. In contrast, the experimental groups (F1, F2, F3) showed an increasing lactic acid concentration during the first 9 days of fermentation, attributed to the ability of lactobacilli to hydrolyze starch in sorghum to produce lactic acid [45,46]. A more rapid increase in lactic acid concentration was observed in F2 and F3 during the later stages of fermentation (Day 9: F2 0.63 ± 0.06 mg/g, F3 0.72 ± 0.05 mg/g; Day 12: F2 1.02 ± 0.08 mg/g, F3 1.13 ± 0.10 mg/g), which is similar to findings reported in previous studies [47]. This trend may be related to *Lactobacillus* becoming the dominant bacterium in the later stages of Chinese baijiu fermentation [13,14]. Compared to the control groups, the total lactic acid concentration in F1, F2, and F3 increased by 43.82, 124.10, and 18.10 times, respectively, indicating that *L. plantarum* significantly contributes to lactic acid production and accumulation during Chinese baijiu fermentation.

As shown in Figure 3B, the ethanol concentration peaked on Day 4 of fermentation in the control groups (CK1: 6.3 ± 0.21 mg/g, CK2: 12.12 ± 0.20 mg/g, CK3: 14.29 ± 0.34 mg/g). However, ethanol was then rapidly consumed, and the ethanol content in CK1, CK2, and CK3 was almost depleted by Day 9 of fermentation. The experimental groups F2 and F3 also showed peaks on Day 4 (F2: 6.51 ± 0.08 mg/g, F3: 5.74 ± 0.02 mg/g), but these peaks were lower than those of the control groups CK2 and CK3. This may be due to the increased lactic acid concentration in F1, F2, and F3, which inhibited the growth of some ethanol-producing microorganisms [48]. Lactic acid bacteria have a regulatory effect on the growth and metabolism of microorganisms in the fermentation system [15,16]. The ethanol concentration in F2 and F3 remained relatively stable after Day 4, ranging from 4 to 6.5 mg/g (Figure 3B).

A previous study on the effect of the microbial community on ethanol concentration during the fermentation of Chinese strong-flavored baijiu found that the ethanol concentration increased to 36.11 ± 0.73 g/L on day 8 and remained unchanged until day 16 [48]. This trend is very similar to the ethanol concentration in F1, F2, and F3 in this study. The metabolic regulation of microorganisms by *L. plantarum* during the fermentation of Chinese baijiu helps maintain the stability of ethanol concentration in the fermentation system, which is crucial for ensuring the consistent quality of Chinese baijiu.

In addition to the comparative analysis of the control and experimental groups, this study revealed an interesting phenomenon. In terms of changes in lactic acid and ethanol content, the lowest concentrations were observed in the fermentation protocols with the lowest microbial diversity (CK1 and F1), and there was a tendency for a rapid decrease in the mid- to late phases of fermentation (Figure 3A,B). Conversely, the highest concentrations were found in the fermentation protocols with the richest microbial diversity (CK3 and F3). This finding may provide new directions and possibilities for targeted regulation and enhancement of Chinese baijiu quality.

### 3.4. Regulation of Organic Acid Species and Concentration by L. plantarum (Except Lactic Acid)

Besides alcohols, acids are also important substrates for the synthesis of esters, which can greatly affect the quality of Chinese baijiu. Currently, the main acids detected in Chinese baijiu are acetic, lactic, propionic, butyric, isobutyric, valeric, isovaleric, capric, and caprylic acids [20]. In this study, we detected four organic acids—acetic acid, propionic acid, butyric acid, and isobutyric acid—in six fermentation protocols. Among these, acetic acid and butyric acid have been shown to have a significant impact on the flavor of Chinese baijiu [1,3].

**Figure 4:**
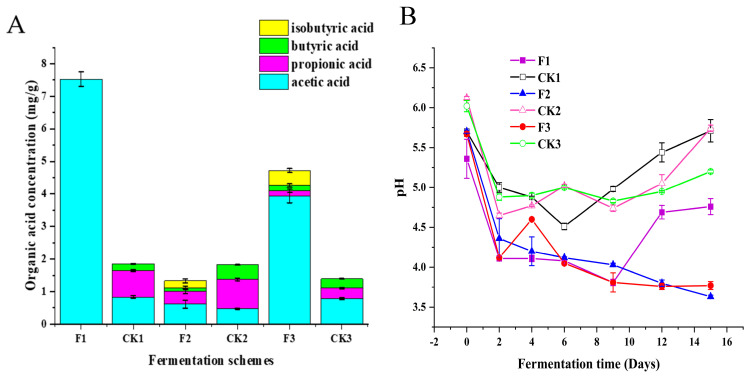
(**A**): Total organic acid concentration (except lactic acid). (**B**): Point line plot of the trend of pH in the experimental (F1, F2, F3) and control (CK1, CK2, CK3) groups during the 15-day fermentation period.

The concentration and pH changes of different organic acids in each fermentation protocol are shown in Figure 4A,B. The total organic acid concentration (excluding lactic acid) in F2 and F3 was significantly higher than in CK2 and CK3 (Figure 4A). The increase in total organic acid concentration (excluding lactic acid) led to a lower pH value in the experimental groups compared to the control groups (Figure 4B).

Previous reports suggested that the pit mud microbial community maintained a stable range for the production and utilization of acetic acid [48]. However, in this study, compared to CK1 and CK3, the total acetic acid concentration in F1 and F3 increased significantly (F1: 7.53 ± 0.29 mg/g, F3: 3.94 ± 0.11 mg/g, CK1: 0.83 ± 0.04 mg/g, CK3: 0.79 ± 0.03 mg/g). The increase in acetic acid concentration in F2 was smaller (F2: 0.61 ± 0.06 mg/g, CK2: 0.47 ± 0.02 mg/g). Isobutyric acid was only detected in F2 and F3 (F2: 0.21 ± 0.04 mg/g, F3: 0.46 ± 0.07 mg/g), and the concentrations of propionic acid and butyric acid showed a slight decrease in F2 and F3.

The total organic acid concentration (excluding lactic acid) in F1 and F3 increased by 3.07 times and 2.40 times, respectively. These changes indicate that the presence of *L. plantarum* in the fermentation process of Chinese baijiu not only increased the total organic acid concentration and reduced the pH of the fermentation environment but also regulated the types and concentrations of organic acids produced.

As shown in Figure 4A, there was little difference in the concentration of organic acids produced by the control groups (CK1, CK2, and CK3). However, after the addition of *L. plantarum*, there was a significant change in the concentration and type of organic acids in F1, F2, and F3. This confirms that *L. plantarum* can greatly influence the interactions between microorganisms and, consequently, the production of organic acids.

### 3.5. L. plantarum Increased the Activity of Glycosylase and Decreased the Activity of α-Amylase

**Figure 5:**
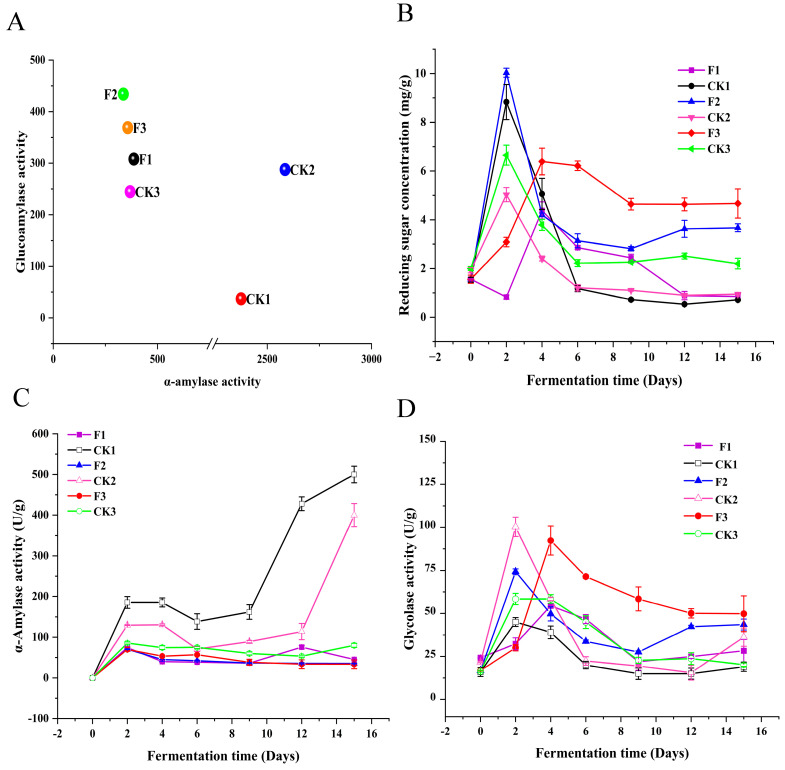
(**A**): Bubble chart of α-amylase (*X*-axis) and glucoamylase (*Y*-axis) activity for the experimental (F1, F2, F3) and control (CK1, CK2, CK3) groups. Points farther from the origin indicate higher enzyme activity. Results are the mean of three replicates. (**B**): Point line plot of reducing sugar concentration trends in experimental (F1, F2, F3) and control (CK1, CK2, CK3) groups over 15 days. (**C**): Point line plot of α-amylase activity trends in experimental (F1, F2, F3) and control (CK1, CK2, CK3) groups over 15 days. (**D**): Point line plot of glucoamylase activity trends in experimental (F1, F2, F3) and control (CK1, CK2, CK3) groups over 15 days.

α-amylase and glucoamylase enzymes primarily degrade starch or polysaccharides into fermentable low molecular weight sugars such as maltose and dextrin, which could subsequently be hydrolyzed to glucose, which in turn would be a precursor to ethanol and other flavor compounds [49,50]. It was evident that α-amylase and glucose amylase play a very important role in the fermentation process of Chinese baijiu. An interesting phenomenon observed from Figure 5A is that the total α-amylase activity in the experimental groups (F1, F2, and F3) and the control groups (CK1, CK2, and CK3) decreased. Specifically, the α-amylase activity in the experimental groups was as follows: F1: 386.50 ± 4.08 U/g, F2: 336.44 ± 3.72 U/g, and F3: 357.14 ± 2.93 U/g, compared to the control groups: CK1: 2374.39 ± 9.25 U/g, CK2: 2585.31 ± 8.47 U/g, and CK3: 368.64 ± 2.23 U/g. Conversely, the total glucoamylase activity increased in the experimental groups: F1: 308.12 ± 2.42 U/g, F2: 434.00 ± 3.11 U/g, and F3: 368.82 ± 3.53 U/g, compared to the control groups: CK1: 36.85 ± 0.93 U/g, CK2: 287.79 ± 1.45 U/g, and CK3: 244.71 ± 1.64 U/g. In the whole fermentation period, the α-amylase and glucoamylase activity on different sampling days also showed the same trend (Figure 5C,D). This phenomenon may be explained by the changes in the activity of the two enzymes during the single strain fermentation process. When the six microorganisms included in the above six fermentation protocols were subjected to single fermentation, only *A. oryzae* and *S. fibuligera* had α-amylase activity (Table 2). Conversely, glucoamylase activity could be detected in all six microorganisms (Table 3). From Table 2 and Table 3, it can be seen that, although *S. fibuligera* has the ability to produce α-amylase, its growth status was insufficient to cause a significant change in α-amylase activity. Therefore, the decrease in α-amylase activity in the three experimental groups was attributed to the weakened growth of *A. oryzae*. While the specific role of *L. plantarum* remains undetermined, it is evident that the addition of *L. plantarum* inhibits the growth of *A. oryzae* and promotes the growth of other glucoamylase-producing microorganisms.

When comparing the reducing sugar concentrations of the experimental groups (F1, F2, and F3) and the control groups (CK1, CK2, and CK3), the reducing sugar concentrations of the three control groups reached their peak on the second day of fermentation (CK1: 8.83 ± 0.72 mg/g, CK2: 5.03 ± 0.29 mg/g, CK3: 6.65 ± 0.41 mg/g). In contrast, the reducing sugar concentrations in the three experimental groups gradually surpassed those of the control groups from the second day onward (Figure 5B). This was because the glucoamylase activity in the experimental groups was lower than that in the control groups during the first two days of fermentation. After the second day, the glucoamylase activity in the experimental groups gradually exceeded that in the control groups (Figure 5D). The increased glucoamylase activity facilitated the conversion of macromolecules into reducing sugars required for microbial growth [49]. *L. plantarum* reduced the activity of α-amylase, increased the activity of glucoamylase, and thereby increased the concentration of reducing sugars.

## 4. Conclusions

In this study, the important metabolites (esters, lactic acid, ethanol, acids, α-amylase, glucoamylase, and reducing sugars) of three experimental groups (F1, F2, and F3) and three control groups (CK1, CK2, and CK3) were compared and analyzed during the fermentation process of Chinese baijiu. It was found that only the experimental groups (F1, F2, and F3) produced ethyl lactate. The addition of *L. plantarum* not only contributed to the production of esters but also influenced the concentration ratios of different esters. The total lactic acid concentrations in the experimental groups (F1, F2, and F3) were 43.82 times, 124.10 times, and 18.10 times higher, respectively, than those in the control groups (CK1, CK2, and CK3). *L. plantarum* helped maintain the stability of ethanol concentration during fermentation, which is of great significance for maintaining the quality of Chinese baijiu. After the addition of *L. plantarum*, the concentrations of acetic acid increased significantly, while the concentrations of propionic acid and butyric acid decreased slightly. Furthermore, the addition of *L. plantarum* resulted in a reduction in α-amylase activity but an increase in glucoamylase activity. Sensory evaluation revealed that *L. plantarum* had a weakening effect on negative odors in the middle and late stages of fermentation and made a significant positive contribution to the aroma and taste.

The results of this study provide a theoretical foundation for the directed regulation of Chinese baijiu flavor using *L. plantarum*. It is possible that other lactic acid bacteria, in addition to *L. plantarum*, may also have different impacts on the biochemical outcomes of fermentation. Furthermore, the interactions between microbial communities are a significant factor influencing baijiu fermentation. However, further research is needed to elucidate these interactions more clearly and to better understand the factors affecting baijiu fermentation.

## Figures and Tables

**Table 1:** Experimental protocol and inoculum amounts.

Category	Protocols	Viable Count of Fungi (×10^7^ CFU/mL)			Viable Count of Bacteria (×10^7^ CFU/mL)		
		*A. oryzae*	*S. cerevisiae*	*S. fibuligera*	*T. actinomycetes*	*B. licheniformis*	*L. plantarum*
		(AO)	(SC)	(SF)	(TA)	(BL)	(LP)
Control group	CK1	1.03 ± 0.04	2.09 ± 0.13	2.27 ± 0.15	—	—	—
	CK2	1.11 ± 0.06	3.09 ± 0.11	2.35 ± 0.13	5.03 ± 0.21	—	—
	CK3	1.08 ± 0.03	2.94 ± 0.09	2.16 ± 0.13	2.33 ± 0.07	3.18 ± 0.19	—
Experimental group	F1	1.23 ± 0.08	3.01 ± 0.15	2.11 ± 0.11	—	—	4.98 ± 0.23
	F2	1.05 ± 0.05	2.92 ± 0.11	2.01 ± 0.09	2.45 ± 0.12	—	3.39 ± 0.18
	F3	1.17 ± 0.07	3.84 ± 0.09	2.59 ± 0.13	2.04 ± 0.06	2.18 ± 0.11	2.45 ± 0.18

Note: “—” indicates that the strain was not added.

**Table 2:** Trends in α-amylase activity during single fermentation of six microorganisms in a 15-day fermentation period.

	Days	0 d	2 d	4 d	6 d	9 d	12 d	15 d
α-Amylase Activity (U/g)								
*A. oryzae* (AO)		ND	3090.85 ± 24.78	3274.11 ± 23.25	3402.53 ± 24.13	25,709.33 ± 22.96	41,713.00 ± 35.51	69,437.64 ± 44.22
*S. cerevisiae* (SC)		ND	ND	ND	ND	ND	ND	ND
*S. fibuligera* (SF)		ND	122.43 ± 3.62	84.02 ± 5.33	60.57 ± 4.36	120.55 ± 6.57	168.81 ± 3.68	225.01 ± 4.85
*T. actinomycetes* (TA)		ND	ND	ND	ND	ND	ND	ND
*B. licheniformis* (BL)		ND	ND	ND	ND	ND	ND	ND
*L. plantarum* (LP)		ND	ND	ND	ND	ND	ND	ND

Note: “ ND ” indicates not detected.

**Table 3:** Trends in glucoamylase activity during single fermentation of six microorganisms in a 15-day fermentation period.

	Day	0 d	2 d	4 d	6 d	9 d	12 d	15 d
Glucoamylase Activity (U/g)								
*A. oryzae* (AO)		ND	820.01 ± 10.21	486.39 ± 16.59	392.92 ± 17.05	1253.16 ± 21.72	2272.21 ± 28.66	4887.91 ± 30.46
*S. cerevisiae* (SC)		ND	9.77 ± 0.31	9.65 ± 1.14	6.49 ± 0.86	5.11 ± 0.43	3.27 ± 0.59	3.91 ± 0.73
*S. fibuligera* (SF)		ND	170.83 ± 3.58	69.23 ± 2.46	41.14 ± 0.43	28.40 ± 0.23	60.06 ± 0.58	99.68 ± 1.21
*T. actinomycetes* (TA)		ND	27.71 ± 0.68	33.13 ± 1.74	28.47 ± 0.71	37.56 ± 0.06	32.95 ± 1.55	38.12 ± 1.83
*B. licheniformis* (BL)		ND	14.36 ± 0.36	21.33 ± 0.67	31.09 ± 0.96	40.83 ± 1.70	49.33 ± 1.18	39.21 ± 1.32
*L. plantarum* (LP)		ND	ND	7.86 ± 0.41	4.71 ± 0.55	5.26 ± 0.31	5.51 ± 0.35	4.43 ± 0.61

Note: “ ND ” indicates not detected.

## Data Availability

The original contributions presented in this study are included in the article. Further inquiries can be directed to the corresponding author.
